# Supplementary material for: Repurposing Penfluridol in Combination with Temozolomide for the Treatment of Glioblastoma
Source: Cancers (Basel). 2019 Sep 5;11(9):1310. doi: 10.3390/cancers11091310 (PMC6770574; doi:10.3390/cancers11091310)
Supplement: Supplementary file 1 [file cancers-11-01310-s001.pdf]

cancers

Supplementary material

Repurposing Penfluridol in Combination with Temozolomide for the Treatment of Glioblastoma

Hyungsin Kim, Kyuha Chong, Byung-Kyu Ryu, Kyung-Jae Park, Mi OK Yu, Jihye Lee, Seok Chung, Seongkyun Choi, Myung-Jin Park, Yong-Gu Chung and Shin-Hyuk Kang

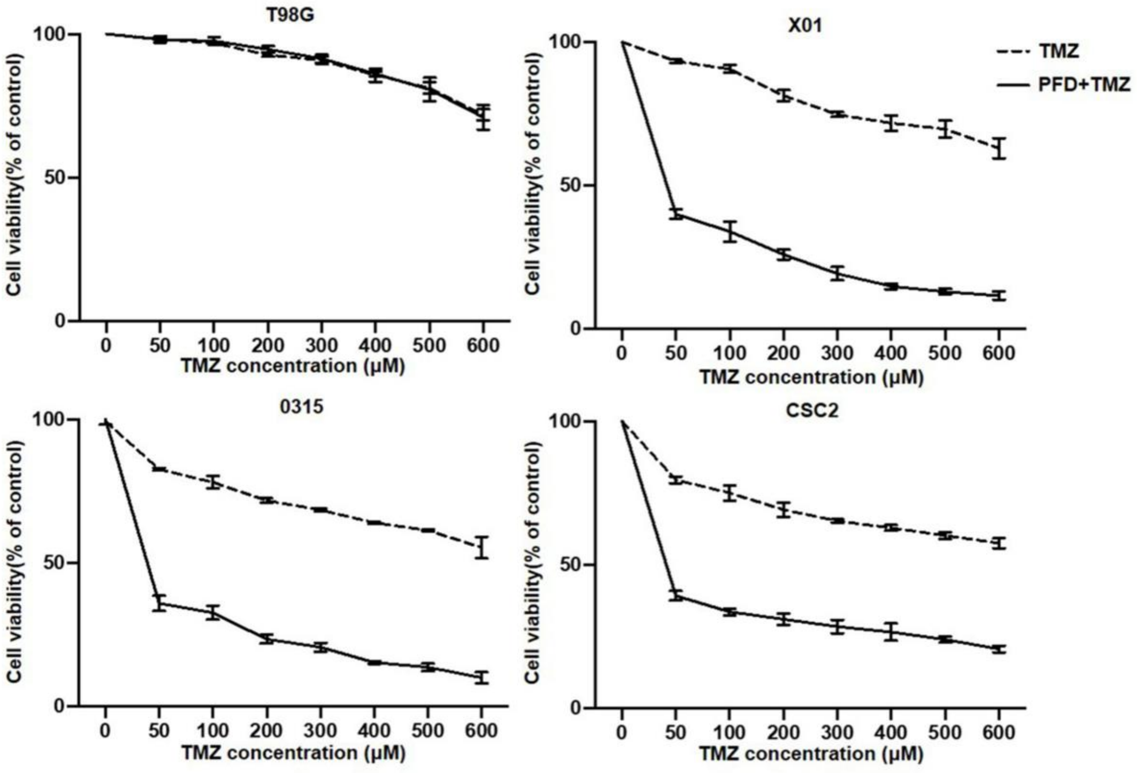

Figure S1. Combined treatment of PFD and TMZ increases the inhibition of cell proliferation in GSCs. T98G glioma cell and X01, 0315, CSC2 GSCs were treated with different concentrations of TMZ or in combination with 2μM PFD. After 48h, cell proliferation was measured by MTT assay. The experiments were repeated three times.

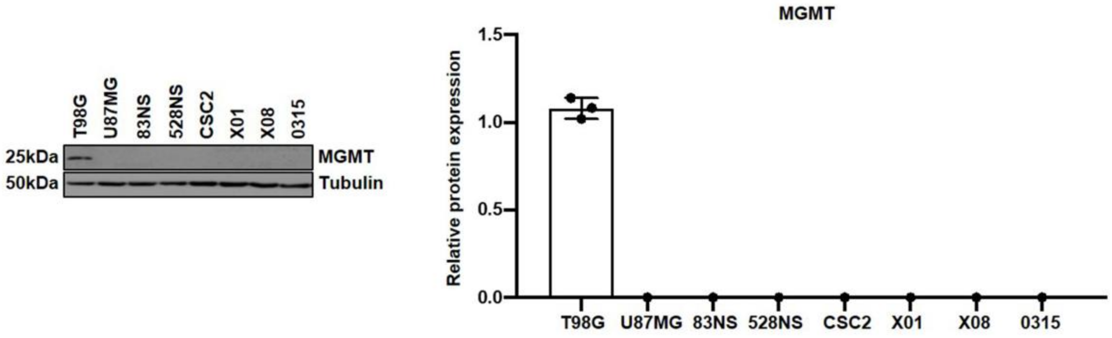

Figure S2. Additional image of Western blot shown in Figure 1A and densitometry ratio of each band. For description of the samples, please refer to Figure legends 1A.

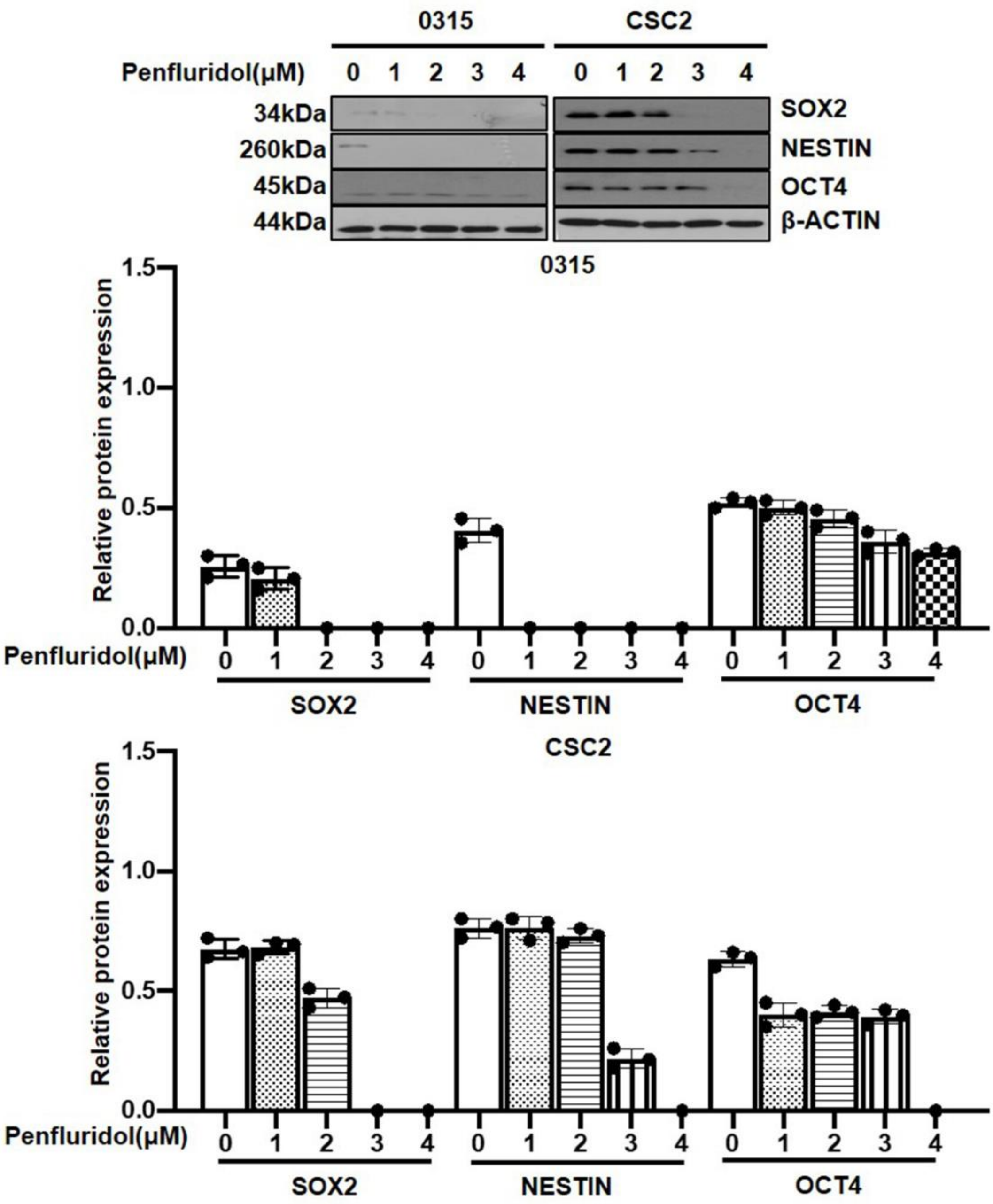

Figure S3. Additional image of Western blot shown in Figure 2E and densitometry ratio of each band. For description of the samples, please refer to Figure legend 2E.

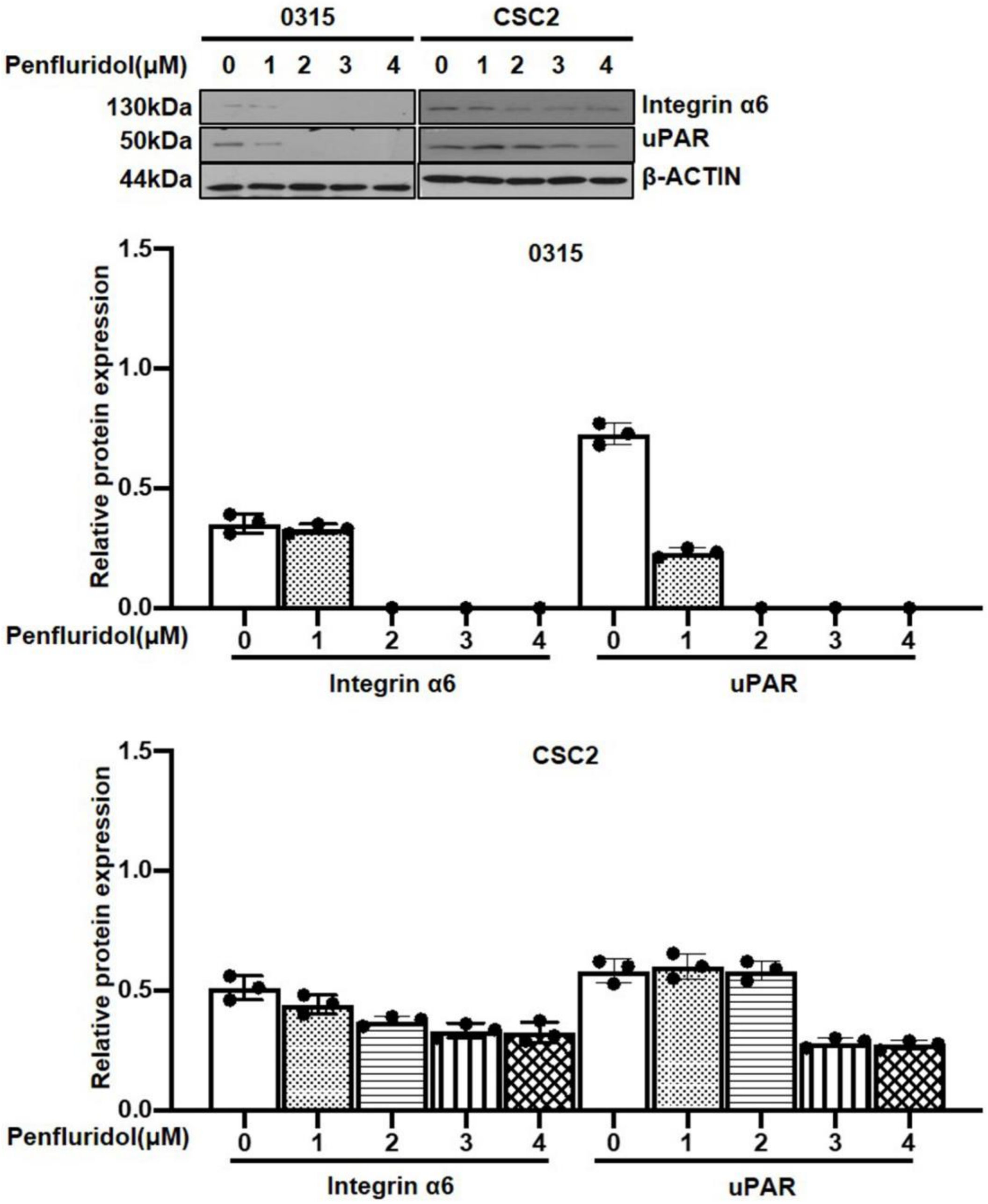

Figure S4. Additional image of Western blot shown in Figure 3E and densitometry ratio of each band. For description of the samples, please refer to Figure legend 3E.

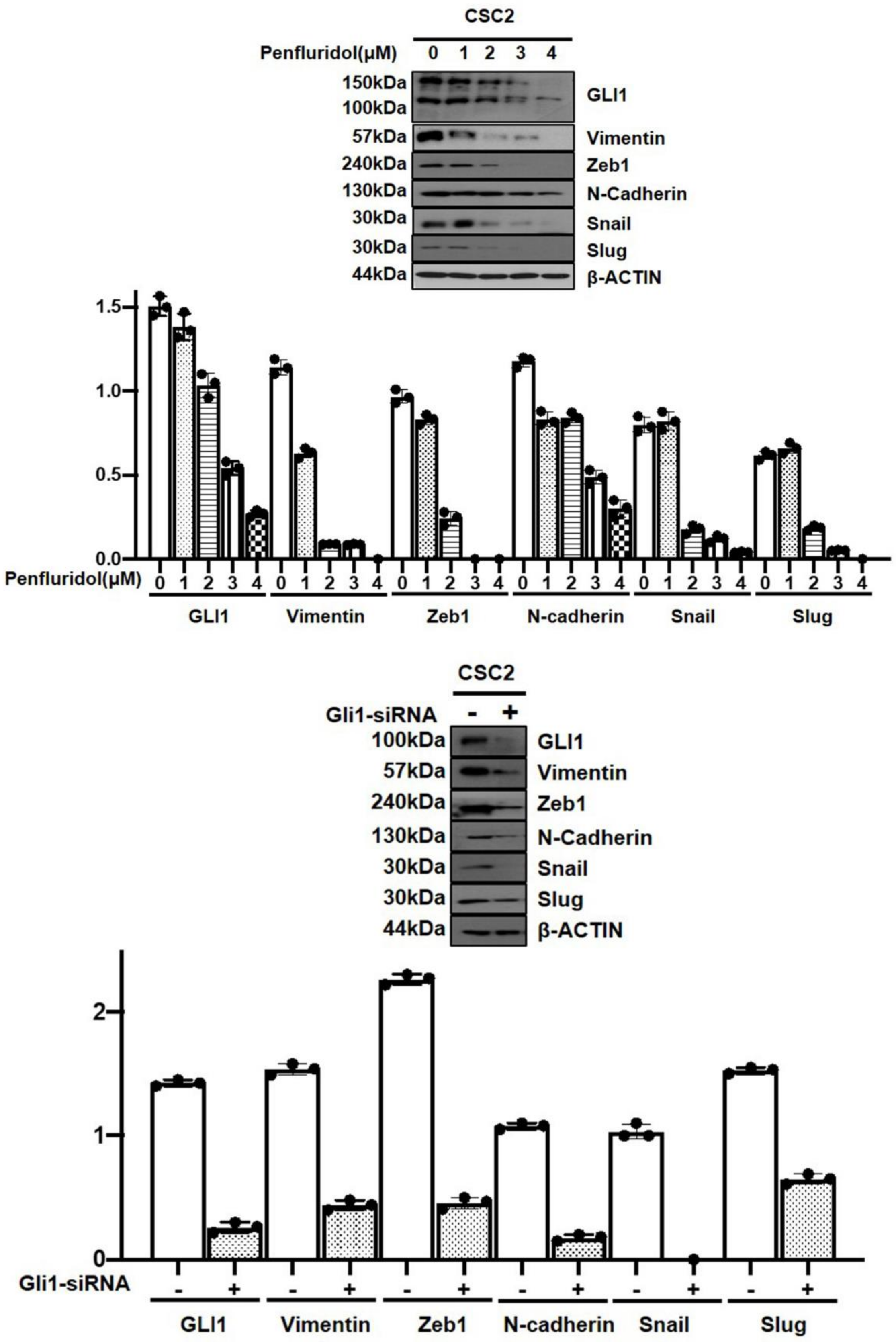

Figure S5. Additional image of Western blot shown in Figure 4B and D and densitometry ratio of each band. For description of the samples, please refer to Figure legends 4B and D.

© 2019 by the authors. Submitted for possible open access publication under the terms and conditions of the Creative Commons Attribution (CC BY) license

(http://creativecommons.org/licenses/by/4.0/).
